# 1,8‐Dihydroxynaphthalene (DHN) melanin provides unequal protection to black fungi *Knufia petricola* and *Cryomyces antarcticus* from UV‐B radiation

**DOI:** 10.1111/1758-2229.70043

**Published:** 2024-11-15

**Authors:** Ilaria Catanzaro, Anna A. Gorbushina, Silvano Onofri, Julia Schumacher

**Affiliations:** ^1^ Bundesanstalt für Materialforschung und ‐prüfung (BAM) Berlin Germany; ^2^ Università degli Studi della Tuscia Viterbo Italy; ^3^ Freie Universität Berlin Germany

## Abstract

Black fungi on rock surfaces endure a spectrum of abiotic stresses, including UV radiation. Their ability to tolerate extreme conditions is attributed to the convergent evolution of adaptive traits, primarily highly melanized cell walls. However, studies on fungal melanins have not provided univocal results on their photoprotective functions. Here, we investigated whether the black fungi *Knufia petricola* and *Cryomyces antarcticus* only use DHN melanin or may employ alternative mechanisms to counteract UV‐induced damage. For this, melanized wild types and non‐melanized Δ*pks1* mutants were exposed to different doses of UV‐B (312 nm) followed by incubation in constant darkness or in light–dark cycles to allow light‐dependent DNA repair by photolyases (photoreactivation). *C. antarcticus* could tolerate higher UV‐B doses but was sensitive to white light, whereas *K. petricola* showed the opposite trend. DHN melanin provided UV‐B protection in *C. antarcticus*, whereas the same pigment or even carotenoids proved ineffective in *K. petricola*. Both fungi demonstrated functional photoreactivation in agreement with the presence of photolyase‐encoding genes. Our findings reveal that although the adaptive trait of DHN melanization commonly occurs across black fungi, it is not equally functional and that there are species‐specific adaptations towards either UV‐induced lesion avoidance or repair strategies.

## Graphical abstract

Wild‐type and non‐melanized Δ*pks1* strains of two rock‐inhabiting fungi were used to study the relevance of 1,8‐dihydroxynaphthalene (DHN) melanin in protection against UV‐B. DHN melanin is essential for the survival of *Cryomyces antarcticus* but not of *Knufia petricola*. Both fungi can repair UV‐damaged DNA through a light‐dependent process known as photoreactivation.

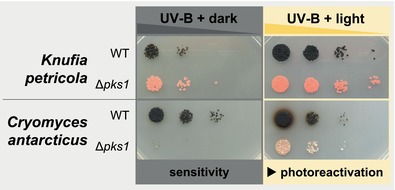

## INTRODUCTION

Solar radiation represents a universal driver for life, with a spectrum spanning from ultraviolet (UV) to infrared. The UV fraction is subdivided into three categories: UV‐C (100–280 nm), UV‐B (280–315 nm) and UV‐A (315–400 nm). The Earth's atmosphere absorbs the most harmful UV wavelengths (<290 nm), preventing them from interacting with the biosphere; however, the incoming UV‐B and UV‐A fractions still cause significant biological effects (Banas et al., 2020; Moan, 2001). UV‐B radiation can induce various chemical alterations in organisms, most notably helix‐distorting lesions in the DNA. These lesions result from the formation of cyclobutane pyrimidine dimers (CPDs) and pyrimidine‐pyrimidone (6‐4) photoproducts (6‐4 PPs) formed by adjacent pyrimidine residues on the same strand (Ravanat et al., 2001). Both CPDs and 6‐4 PPs are highly cytotoxic and mutagenic. Single‐strand breaks (SSBs) and double‐strand breaks (DSBs) in the DNA may also occur (Rastogi et al., 2010). UV‐A radiation mainly leads to the accumulation of reactive oxygen species (ROS), which indirectly damage DNA, proteins, and lipids (Ravanat et al., 2001; Sinha & Hader, 2002). Once organisms are able to cope with the deleterious effects of sunlight, they can use it as an energy source in terms of photosynthesis or heat production, and/or as a signal to regulate their metabolism, development and behaviour, and to run a circadian clock to anticipate daily upcoming changes (Seemann et al., 1979; Dunlap, 1999).

Many organisms synthesize metabolites that may function as a first line of defence against UV radiation by mitigating the consequences of habitual exposure to intense sunlight (Kreusch & Duarte, 2021). Melanins constitute a heterogenous group of macromolecules derived from the oxidative polymerization of different phenolic precursors. Their broad‐spectrum absorption, especially in the UV range, and antioxidant properties support their protective role against UV radiation (Cordero & Casadevall, 2020). Several fungi produce the black 1,8‐dihydroxynaphthalene (DHN) melanin, which derives from the extracellular polymerization of DHN. Several filamentous Ascomycetes including *Botrytis cinerea* produce DHN melanin in a spatiotemporal manner, such as in stressed mycelia and reproductive structures (Schumacher, 2016). In contrast, the vegetative cells of microcolonial black fungi, commonly found in various habitats exposed to high solar irradiance, for example, on rock surfaces in cold and hot deserts, are always melanized (Liu et al., 2021; Tesei, 2022). Orange to reddish carotenoids are tetraterpenoids that act as antioxidants and mitigate UV‐induced stress primarily by quenching ROS (Sandmann, 2019). They are produced by fungi in a spatiotemporal manner or constitutively (Avalos et al., 2017; Chen et al., 2014; Flieger et al., 2018). Mycosporines are water soluble, colourless natural products with an absorption band between 309 and 362 nm. They dissipate energy as heat without generating ROS and are discussed as multipurpose secondary metabolites (Gao & Garcia‐Pichel, 2011; Geraldes & Pinto, 2021; Oren & Gunde‐Cimerman, 2007). The production of mycosporines is common to cyanobacteria, aquatic yeasts, and rock‐inhabiting and halophilic black fungi (Kogej et al., 2006; Libkind et al., 2006; Volkmann et al., 2003; Volkmann & Gorbushina, 2006).

The second line of defence against harmful UV radiation encompasses the enzymatic detoxification of ROS and the repair of damaged molecules. UV‐induced lesions and breaks in the DNA need to be repaired before replication occurs to ensure that the genetic information remains intact (Sinha & Hader, 2002; Wong et al., 2019). CPDs and 6‐4 PPs are cleaved by photolyases to restore the original base pairing, in a light‐dependent process called photorepair or photoreactivation (Essen & Klar, 2006). These can also be repaired independently of light by nucleotide excision repair (NER) or alternative excision repair (AER), while other UV lesions such as oxidized purines, SSBs, and DSBs are repaired by base excision repair (BER), non‐homologous end‐joining (NHEJ), or homologous recombination (HR) (Chatterjee & Walker, 2017; Strzalka et al., 2020; Yasui, 2013). Like bacteria and plants, most fungi use photoreactivation to cope with UV‐induced DNA lesions, but the number of photolyases differs among Ascomycetes (Schumacher & Gorbushina, 2020).

Due to their worldwide distribution in various sunlight‐flooded environments, microcolonial black fungi are thought to employ diverse protective mechanisms to mitigate and/or repair UV‐induced damage. However, detailed studies employing deletion mutants to decipher the mechanisms in black fungi are scarce due to the difficulty of transforming them.

The aim of this study was to combine, for the first time, two black fungi from different lineages and their non‐melanized (Δ*pks1*) mutants and to test them in the same experimental set‐up to investigate the role of protective pigments and photoreactivation in surviving UV stress. *Knufia petricola* (Eurotiomycetes), colonizing exposed rocks and human‐made structures (Gorbushina et al., 2008), has become a model for exploring the physiology and lifestyle of rock‐inhabiting fungi due to its moderate growth rate and available tools for genetic engineering (Erdmann et al., 2022; Nai et al., 2013; Voigt et al., 2020). *Cryomyces antarcticus* (Dothideomycetes), a cryptoendolithic fungus (Selbmann et al., 2005), is known to withstand many extreme conditions, including UV and ionizing radiation (Pacelli et al., 2021; Selbmann et al., 2018), and has long been used as a test organism in astrobiological research on the limits of life (Onofri et al., 2020; Simões et al., 2023). The results indicate that *C. antarcticus* tolerates higher doses of UV‐B radiation than *K. petricola*, that DHN melanin is important for survival of *C. antarcticus* but not of *K. petricola*, and that the survival rates of both fungi are increased by incubation in visible light upon UV‐B exposure. Implications for species‐specific adaptations between these black fungi are discussed.

## EXPERIMENTAL PROCEDURES

### Fungal strains and cultivation

*K. petricola* and *C. antarcticus* wild‐type (WT) and mutant strains listed in Table 1 were cultivated on malt extract agars, that is, on MEA [malt extract agar: 2.0% malt extract (Carl Roth GmbH+ Co. KG), 0.1% peptone from casein (Carl Roth GmbH+ Co. KG), 2.0% D‐glucose, 2.0% Kobe agar (AppliChem GmbH)] or MEAV [malt extract agar variant: 3.0% malt extract (Carl Roth GmbH+ Co. KG), 1.5% bacteriological agar (AppliChem GmbH)], on SDNG [synthetic‐defined nitrate glucose medium: 0.17% Difco yeast nitrogen base without amino acids and ammonium sulfate (BD Biosciences), 0.3% NaNO_3_, 2.0% D‐glucose, 2.0% Kobe agar], and on WA [water agar: 2.0% Kobe agar]. Biomass from one‐week‐old *K. petricola* or eight‐week‐old *C. antarcticus* MEA cultures was homogenized in 1× phosphate‐buffered saline [PBS: 137 mM NaCl, 2.7 mM KCl, 10 mM Na_2_HPO_4_, 1.8 mM KH_2_PO_4_, pH 7.4] using glass beads and a Retsch mixer mill (10 min at 30 Hz). 20 mL of MEB [2.0% malt extract, 0.1% peptone from casein, 2.0% D‐glucose] were inoculated with 200 μL of cell suspensions and incubated in the dark on a rotary shaker at 100 rpm (*K. petricola*) or shaken 1 min/week (*C. antarcticus*). Agar cultures were incubated either in constant darkness (DD) or under a 12 h white light (400–700 nm)/12 h dark cycle (LD), with *K. petricola* strains at 25°C (OSRAM DULUX L cool white, 116 μmol photons/m^2^/s) and *C. antarcticus* strains at 15°C (Philips F32T8/TL741 cool white, 118 μmol photons/m^2^/s).

**TABLE 1:** Fungal strains used in this study.

Strain name [BAM ID]	Genotype	Reference
*Knufia petricola* A95 (CBS 123.872)	WT	Voigt et al. (2020)
Δ*kppks1* [KP‐0037; T3]	A95, Δ*pks1* [(T*niaD*::*hph*::P*trpC*)]	Voigt et al. (2020)
Δ*kpphs1* [KP‐0054; T3]	A95, Δ*phs1* [(T*niaD*::*nat1*::P*trpC*)]	Voigt et al. (2020)
Δ*kppks1*/Δ*kpphs1* [KP‐0083; T4]	A95, Δ*pks1* [(T*niaD*::*nat1*::P*trpC*)], Δ*phs1* [(T*niaD*::*hph*::P*trpC*)]	Voigt et al. (2020)
*Cryomyces antarcticus* MNA‐CCFEE 515	WT	Selbmann et al. (2005)
Δ*capks1* [CA‐0002; T10, T17]	MNA‐CCFEE 515, Δ*pks1* [(T*niaD*::*hph*::P*trpC*)]	Catanzaro et al. (2024)

### UV‐B survival assessment

Biomass harvested from three‐day‐old *K. petricola* or four‐week‐old *C. antarcticus* liquid cultures was homogenized in a stainless‐steel beaker with seven stainless‐steel beads using a Retsch mixer mill (2.5 min at 30 Hz). Cell suspensions were washed with Milli‐Q water and centrifuged at 3000 × *g* for 5 min. Titers of colony‐forming units (CFU) were determined using a Thoma cell counting chamber and adjusted with 1× PBS to 2.5 × 10^3^ CFU/mL for plating and 1 × 10^8^ CFU/mL for dropping. For survival assays, 200 μL of cell suspension (approx. 5.0 × 10^2^ CFU) were spread evenly on agar using 10 glass beads (3–5 mm). For drop assays, serial dilutions down to 1 × 10^3^ CFU/mL were prepared, and 10 μL (1 × 10^6^ to 1 × 10^1^ CFU) were dropped on agar. Inoculated Petri dishes without lids were exposed to medium wavelength ultraviolet light (UV‐B, 312 nm) in a Bio‐Link BLX crosslinker (VILBER). A range of preset UV‐B doses from 0.1 up to 2.3 J/m^2^ were delivered at varying exposure times, calculating an irradiance of approx. 3400 μW/cm^2^. Irradiated Petri dishes were covered with lids and either immediately wrapped in aluminium foil and incubated in DD or exposed to LD conditions to allow photoreactivation. Survival rates (%) were determined for each strain tested and UV‐B dose by calculating the average number of colonies on irradiated replicates compared to that on control replicates, with colony counts performed after one (for *K. petricola* strains) or eight (for *C. antarcticus* strains) weeks of incubation in DD. Survival assays included a minimum of three biological replicates per strain tested and were performed at three independent times (*n* ≥ 9). Lethal dose values, defined as the radiation dose required to inactivate 50% (LD_50_), 75% (LD_75_), or 90% (LD_90_) of the microbial population, were estimated for the WT strains from their dose–response curves.

### Standard molecular methods

Genomic DNA from *K. petricola* strains was extracted as described previously (Voigt et al., 2020). DNA and the 1 kb Plus DNA Ladder (New England Biolabs, NEB), mixed with Midori Green Direct (Biozym Scientific GmbH), were separated in 1% agarose gels in 0.5% Tris‐acetate‐EDTA (TAE) buffer in a Mupid exU gel electrophoresis chamber and visualized with a ChemiDoc XRS+ Imager equipped with Image Lab 6.0.1 (Bio‐Rad Laboratories Inc.). Regions of interest were amplified with the Q5 High‐Fidelity DNA Polymerase (New England Biolabs, NEB) and primers listed in Table S1. Amplicons were purified with the Monarch PCR & DNA Cleanup Kit (NEB) and sequenced with appropriate primers at Eurofins Genomics.

### Bioinformatics analyses

*C. antarcticus* nucleotide and protein sequences of putative photoreceptors and light‐responsive transcription factors listed in Table S2 were extracted from the *C. antarcticus* CBS 116301 v3.0 database (https://mycocosm.jgi.doe.gov/Cryan3) at the Joint Genome Institute. Muscle alignments for calculating amino acid identities in percent and phylogenetic trees were generated using Geneious Prime 2023.2.1 (Biomatters Ltd.). Sanger sequencing reads were mapped onto the respective *K. petricola* sequences using SnapGene v. 4.0.8 (GSL Biotech LLC).

## RESULTS

### K. petricola *grows faster than* C. antarcticus *even at 15°C and in light–dark cycles*

*K. petricola* strain A95 (*Kp* WT:A95) was isolated from a marble monument in Athens (Greece) (Gorbushina et al., 2008) and is routinely incubated at 25°C in the dark. *C. antarcticus* strain MNA‐CCFEE 515 (*Ca* WT:515) was isolated from Antarctic sandstone at Linnaeus Terrace (McMurdo Dry Valleys, Southern Victoria Land) and is cultivated in darkness at 15°C, which allows for optimum growth rates (Selbmann et al., 2005).

**FIGURE 1:**
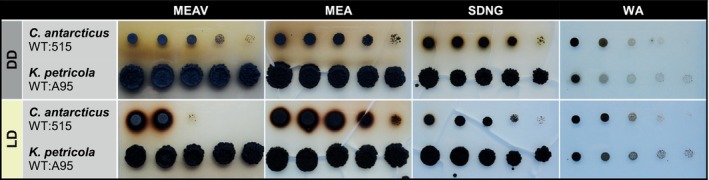
Growth and pigmentation of *Cryomyces antarcticus* and *Knufia petricola* under different conditions. Four different solid media were inoculated with cell suspensions (10^6^, 10^5^, 10^4^, 10^3^, and 10^2^ colony forming units per droplet) from wild‐type strains *C. antarcticus* MNA‐CCFEE 515 (*Ca* WT:515) and *K. petricola* A95 (*Kp* WT:A95), and incubated for 8 weeks at 15°C in either constant darkness (DD) or 12 h white light/12 h dark cycles (LD). Full media: MEA, malt extract agar; MEAV, malt extract agar variant. Minimal media: SDNG, synthetic‐defined nitrate‐glucose medium; WA, water agar.

To compare the growth phenotype of the two wild types under different nutrient and light conditions, cell suspensions were dropped onto solidified media and incubated at 15°C in either complete darkness (DD) or 12 h light/12 h dark cycles (LD). Increased biomass after 8 weeks of incubation was used as a proxy for the strain growth rate. The media were: (1) MEA, a nutrient‐rich medium with glucose and peptone as carbon and nitrogen sources routinely used for the cultivation of *K. petricola*; (2) MEAV, an alternative MEA lacking glucose and peptone used for the cultivation of Antarctic fungi (Pacelli et al., 2020a); (3) SDNG, a synthetic medium containing vitamins, trace elements, nitrate and glucose used for transformant selection; and (4) WA, a minimalist medium lacking all nutrients (Figure 1 and Table 1). After 8 weeks under DD conditions, both strains exhibited growth on all substrates with the typical compact colonies of rock‐inhabiting fungi, with the highest biomass produced on MEA. Notably, *Kp* WT:A95 was able to thrive at the low temperature of 15°C and maintained a faster growth rate than *Ca* WT:515 on all nutrient‐containing media and independently of light conditions. However, both fungi showed equally slow growth on WA. *Ca* WT:515 showed instead altered growth rates, especially on MEAV, depending on the light condition. The colonies of *Ca* WT:515 secreted dark metabolites in nutrient‐rich media. Growth rates of *Ca* WT:515, but not of *Kp* WT:A95, were reduced in the presence of light (118 μmol photons/m^2^/s).

### C. antarcticus *can tolerate higher UV‐B doses than* K. petricola

**FIGURE 2:**
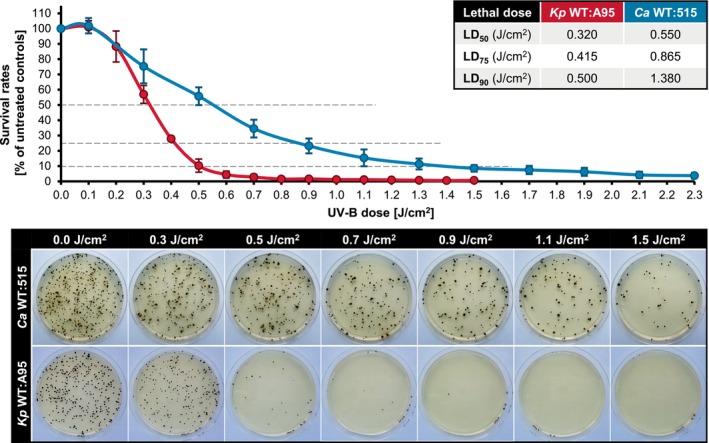
*Cryomyces antarcticus* cells survive higher doses of UV‐B irradiation than *Knufia petricola* cells. Approx. 5 × 10^2^ colony forming units of *C. antarcticus* MNA‐CCFEE 515 (*Ca* WT:515) and *K. petricola* A95 (*Kp* WT:A95) were spread onto MEA (malt extract agar) [at least three plates per strain and UV‐B dose (J/cm^2^) as technical replicates], treated with the indicated UV‐B doses (312 nm), and incubated in constant darkness for 1 week at 25°C (*Kp* WT:A95) or 8 weeks at 15°C (*Ca* WT:515). Relative mean values and standard deviations of survival rates (colonies on treated plates/colonies on untreated controls) were calculated from three (*Ca* WT:515) and four (*Kp* WT:A95) biological replicates. LD_50,75,90_—lethal dose, required to kill 50%, 75%, or 90% of the cells. Pictures of representative plates are shown.

To assess the impact of UV‐B radiation on the viability of both *K. petricola* and *C. antarcticus* wild types, MEA plates were inoculated with approx. 500 CFU to ensure a uniform cell distribution and prevent multi‐layer shielding effects. The inoculated plates were exposed to incremental doses of UV‐B radiation, ranging from 0.1 to 1.5 J/cm^2^ for *Kp* WT:A95 and from 0.1 to 2.3 J/cm^2^ for *Ca* WT:515. Following irradiation, the plates were kept in the dark at the optimum growth temperature for each strain. The results were plotted as dose–response curves, revealing different survival capacities of the two fungi to UV‐B irradiation (Figure 2). *Kp* WT:A95 maintained colony growth only at lower UV‐B doses, with survival rates halved at 0.320 J/cm^2^ (LD_50_) and falling to 10% at 0.500 J/cm^2^ (LD_90_). In contrast, *Ca* WT:515 exhibited a slower decline in survival rates, which halved at the dose of 0.550 J/cm^2^ (LD_50_). Sustained growth was observed up to 1.380 J/cm^2^ (LD_90_). By maintaining significantly higher LD_50_ (0.550 vs. 0.320 J/cm^2^) and LD_90_ (1.380 vs. 0.500 J/cm^2^) values, *C. antarcticus* demonstrated higher tolerance to UV‐B irradiation than *K. petricola*.

### *Neither DHN melanin nor carotenoids are involved in the UV‐B tolerance of* K. petricola

*K. petricola* produces at least three photoprotective compounds. DHN melanin is deposited on the outer surface of the cell wall, carotenoids are integrated into the membrane, and mycosporines are considered to accumulate in the cytosol (Breitenbach et al., 2022; Flieger et al., 2018; Volkmann & Gorbushina, 2006). Since the pathways for the synthesis of DHN melanin and carotenoids are known, pigment‐deficient *K. petricola* mutants lacking any intermediates were generated by deleting the key enzyme‐encoding genes. Thus, deletion of *kppks1*, which encodes the single polyketide synthase of *K. petricola*, results in non‐melanized mutants with pinkish pigmentation due to the now visible carotenoids. Deletion of *kpphs1*, which encodes the bifunctional phytoene synthase/lycopene cyclase, results in carotenoid‐deficient mutants that exhibit either a black pigmentation in a wild‐type background or an albino phenotype in a Δ*kppks1* background. All mutants exhibit wild‐type‐like growth rates under non‐stress conditions (Voigt et al., 2020).

**FIGURE 3:**
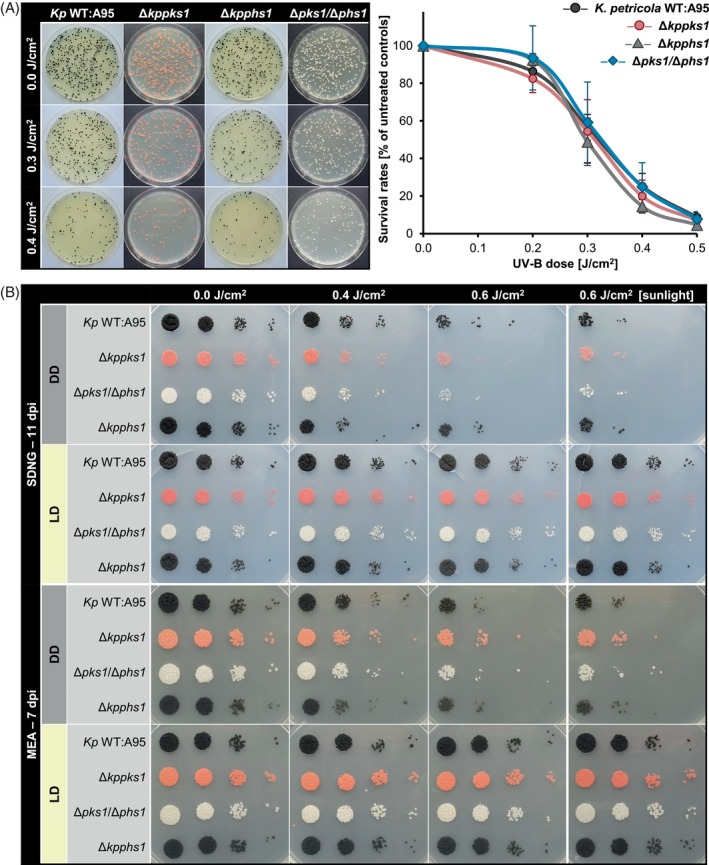
Neither DHN melanin nor carotenoids protect *Knufia petricola* cells from UV‐B irradiation. (A) Pigment‐deficient mutants of *K. petricola* are sensitive to UV‐B as the wild type A95. Cells [5 × 10^2^ colony forming units (CFU) per MEA (malt extract agar) plate, four plates per strain and UV‐B dose (J/cm^2^) as technical replicates] of *Kp* WT:A95 and three pigment‐deficient mutants were exposed to the indicated UV‐B doses and incubated for 1 week at 25°C in the dark. Survival rates were calculated from four biological replicates. Representative plates are shown on the left. (B) White light upon UV‐B fully restores survival of all *K. petricola* strains indicating efficient photoreactivation. Cells (10^4^–10^1^ CFU) of the *K. petricola* strains on MEA or SDNG (synthetic‐defined nitrate glucose) agar were exposed to UV‐B. Plates on the left (0.0, 0.4, and 0.6 J/cm^2^) were incubated at 25°C in either constant darkness (DD) or 12 h white light/12 h dark cycles (LD) in a climate chamber. Plates on the right (column 0.6 J/cm^2^ [sunlight]) were incubated either in DD or on a window board in summer [sunlight]. dpi, days post inoculation.

An assay was performed with the melanized *Kp* WT:A95 and three mutants lacking the ability to synthesize either DHN melanin (Δ*kppks1*), carotenoids (Δ*kpphs1*), or both pigments (Δ*kppks1*/Δ*kpphs1*). UV‐B survival rates were determined for 0.2, 0.3, 0.4, and 0.5 J/cm^2^ for each strain (Figure 3A). *Kp* WT:A95 maintained survival rates consistent with the previous experiment, and all three mutants exhibited similar responses to the wild type. At 0.3 J/cm^2^, where *Kp* WT:A95 showed a nearly halved survival rate (56.6 ± 6.9%), Δ*kppks1*, Δ*kpphs1* and Δ*kppks1*/Δ*kpphs1* showed comparable survival rates with 54.4 ± 16.9%, 48.8 ± 12.6% and 59.2 ± 21.5%, respectively. All pigment‐deficient strains demonstrated similar sensitivity to UV‐B irradiation, resembling survival thresholds of the wild‐type strain up to the highest dose tested of 0.5 J/cm^2^. This demonstrated that the absence of either DHN melanin, carotenoids, or both pigments has no significant impact on the UV‐B tolerance of *K. petricola*.

### *DHN melanin significantly contributes to the UV‐B tolerance of* C. antarcticus

Research on *C. antarcticus* has focused on melanin—chemical studies suggested that the fungus produces both DHN and L‐3,4 dihydroxyphenylalanine (L‐DOPA) melanin (Pacelli et al., 2020b)—rather than on further photoprotective compounds. Due to its slow growth rate, the generation of mutants in *C. antarcticus* is time‐consuming but feasible, as recently shown. As the first gene assumed to be essential for DHN melanin synthesis, *capks1* has been successfully deleted. *Capks1* deletion mutants are non‐melanized and have equal growth rates compared to the wild type. The slight pink pigmentation further suggests that carotenoids are produced; however, Δ*capks1/*Δ*caphs1* mutants are not available (Catanzaro et al., 2024).

**FIGURE 4:**
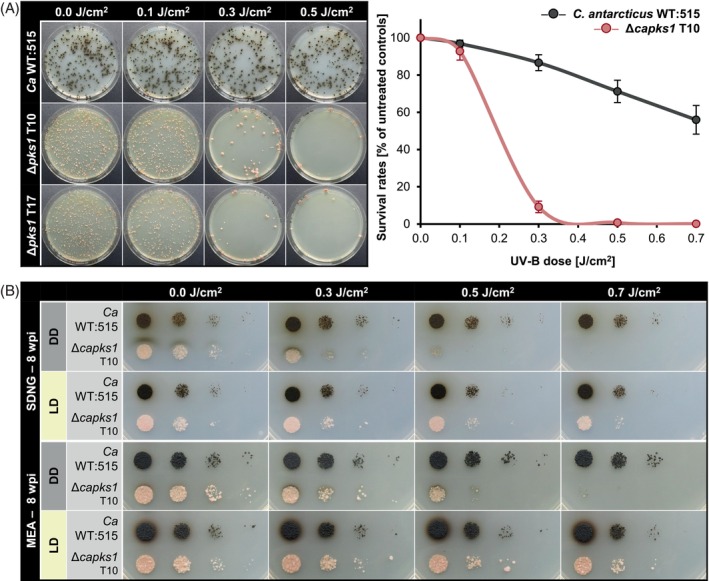
DHN melanin protects *Cryomyces antarcticus* cells from UV‐B irradiation. (A) The non‐melanized *C. antarcticus* Δ*pks1* mutant is hypersensitive to UV‐B. Cells [5 × 10^2^ colony forming units (CFU) on MEA (malt extract agar), four plates per strain and UV‐B dose (J/cm^2^) as technical replicates] of the *C. antarcticus* wild type (*Ca* WT:515) and two melanin‐deficient mutants (Δ*capks1* T10, T17) were exposed to the indicated UV‐B doses and incubated at 15°C in the dark. On the left: representative pictures were taken after 15 weeks. On the right: relative survival rates of *Ca* WT:515 and the Δ*capks1* mutant T10 were calculated from four biological replicates. Colonies were counted after 8 weeks of incubation. (B) White light upon UV‐B exposure increases the survival rate of the non‐melanized mutant, but not that of the wild type. Cells (10^4^–10^1^ CFU) of the wild type (*Ca* WT:515) and the Δ*capks1* mutant (T10) were dropped onto MEA or SDNG (synthetic‐defined nitrate glucose) agar and exposed to UV‐B and incubated at 15°C in either constant darkness (DD) or 12 h white light/12 h dark cycles (LD). Pictures were taken at 8 weeks post inoculation (wpi).

In a first UV‐B survival assay, the melanized *Ca* WT:515 and two independent Δ*capks1* mutants (T10 and T17) were tested. Survival rates were determined for each strain at UV‐B doses of 0.1, 0.3, 0.5, and 0.7 J/cm^2^ (Figure 4A). As both mutants yielded similar results, *Ca* WT:515 and Δ*capks1* T10 were used for the three additional experiments to obtain four biological replicates. While *Ca* WT:515 showed a gradual decrease in cell survival, maintaining a survival rate of 55.9 ± 7.7% at the highest dose of 0.7 J/cm^2^, the Δ*capks1* mutant showed a steep decline from lower doses, with a tenfold decrease in survival from 0.1 J/cm^2^ (92.7 ± 4.7%) to 0.3 J/cm^2^ (9.2 ± 3.1%). The Δ*capks1* mutant was also more sensitive to UV‐B than the wild‐type and mutant strains of *K. petricola*, showing survival rates of about 50% at the same dose of 0.3 J/cm^2^. In sum, the higher sensitivity of the non‐melanized Δ*capks1* compared to the melanized *Ca* WT:515 demonstrated that DHN melanin protects *C. antarcticus* from UV‐B irradiation.

### *UV‐B exposure resulted in non‐melanized mutants of* K. petricola *but not of* C. antarcticus

**FIGURE 5:**
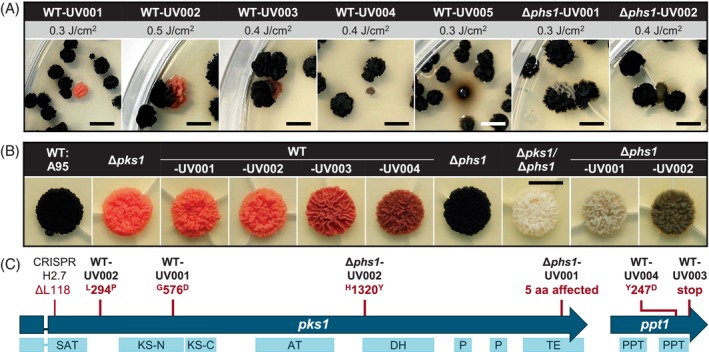
Exposure of *Knufia petricola* to UV‐B resulted in mutants with altered melanization patterns. (A) Differentially pigmented colonies derived from treatment of wild type and carotenoid‐deficient cells with UV‐B. Plates from the experiments shown in Figure 3A [UV‐B doses (J/cm^2^) are shown above] were photographed after 2 months of incubation at 25°C. Cells from colonies with altered pigmentation were transferred to MEA (malt extract agar). WT‐UV001 to WT‐UV004 derived from WT, Δ*phs1*‐UV001 to Δ*phs1*‐UV002 derived from Δ*phs1*. Scale bars: 5 mm. (B) The isolated UV mutants likely produce different amounts of DHN melanin. Cell suspensions of the indicated strains were dropped onto MEA and cultivated for 12 days at 25°C in the dark. Scale bar: 1 cm. (C) Sequencing revealed non‐silent mutations in *kppks1* and *kpppt1*. The coding regions of *kppks1* and *kpppt1* were amplified and sequenced. The identified mutations were mapped to the genes. The conserved protein domains are shown below: AT, acyl transferase [PF00698]; DH, dehydratase [PF14765]; KS, β‐ketoacyl synthase [PF00109, PF02801]; P, phosphopantetheine attachment site [PF00550]; PPT, 4′‐phosphopantetheinyl transferase [IPR037143]; SAT, starter unit: ACP transacylase [PF16073]; TE, thioesterase [PF00975]. The 3‐bp‐long (ΔL118) deletion in the CRISPR mutant H2.7 was identified in a previous study (Voigt et al., 2020).

The experiments carried out resulted in some *K. petricola* colonies with altered pigmentation (Figure 5A). In contrast, no such colonies were observed for *C. antarcticus* in any of the test plates. Obtained mutants from *Kp* WT:A95 formed pink to dark pink colonies (WT‐UV001–UV004) or showed severely reduced growth accompanied by the secretion of brownish pigments (WT‐UV005). Mutants derived from the carotenoid‐deficient Δ*kpphs1* mutant exhibited a light to dark grey pigmentation (Δ*phs1*‐UV001 and Δ*phs1*‐UV002). The mutants exhibiting reduced melanization were isolated, their DNA extracted and used for the amplification of the *kppks1* coding region (Figure 5B). Sequencing of the amplicons revealed point mutations, all non‐silent, in WT‐UV001 (^G^576^D^), WT‐UV002 (^L^294^P^), Δ*phs1*‐UV002 (^H^1320^Y^), and a deletion/mutation affecting five aa in the C‐terminus in Δ*phs1*‐UV001 (Figure 5C). No mutations in *kppks1* were identified for WT‐UV003 and WT‐UV004. Therefore, *kpppt1*, which encodes a 4′‐phosphopantetheinyl transferase for post‐translational activation of polyketide synthases (PKS), was amplified. Sequencing identified non‐silent point mutations, that is, ^Y^247^D^ in WT‐UV003 and a premature stop codon in WT‐UV004, resulting in a protein lacking the last 58 aa. This demonstrated the requirement of *kpppt1* for DHN melanogenesis. The producibility of mutants with altered pigmentation from *Kp* WT:A95 in contrast to *Ca* WT:515 is consistent with the higher sensitivity to UV‐B/mutation rate of *K. petricola*.

### Light mediates repair of UV‐B damaged DNA in both species

Since photolyases are light‐driven enzymes, the total photoreactivation capacity of an organism can be elucidated by exposing UV‐B‐treated cells to white light. In this regard, two solidified media (MEA and SDNG) were inoculated with cell suspensions (10^4^ to 10^1^ CFU per drop) of *K. petricola* or *C. antarcticus* strains, irradiated with different doses of UV‐B and then incubated in LD to allow photoreactivation and in DD as a control. Plates inoculated with the *K. petricola* wild type and pigment‐deficient mutants were exposed either to 116 μmol photons/m^2^/s at 25°C in a climate chamber or to sunlight and ambient temperature on a window board (summer) (Figure 3B). In both setups, white light efficiently mediated photoreactivation in the four *K. petricola* strains, as growth was rescued by light on both media even after exposure to the highest UV‐B dose (0.6 J/cm^2^). UV‐treated cells developed colonies in the light, as did untreated controls. These experiments further demonstrated that the pigmented and pigment‐deficient strains of *K. petricola* grow equally well in light and dark, producing similar amounts of carotenoids. For *C. antarcticus*, the wild type and the non‐melanized (Δ*capks1*) mutant were tested (Figure 4B). The drop assay confirmed the hypersensitivity of Δ*capks1* to low doses of UV‐B. Incubation in light increased the survival of the mutant after UV‐B exposure on both media almost to that of the melanized *Ca* WT:515. However, the growth capacity of both strains never returned to that of the untreated controls.

Taken together, exposure to white light after UV‐B treatment increased the survival capacities of both species, indicating that they can employ photoreactivation to mitigate UV‐B‐induced damage – when light is available.

## DISCUSSION

*K. petricola* and *C. antarcticus* are black fungi from different lineages of the Ascomycota living in lithic microbial communities. *K. petricola* is frequently found in subaerial biofilms from Mediterranean regions (Gorbushina & Broughton, 2009; Marvasi et al., 2012; Sert et al., 2007; Wollenzien et al., 1997). Recently, this species has also been reported from the Antarctic (Selbmann et al., 2021). In contrast, the distribution of *C. antarcticus* is restricted to cryptoendolithic communities in Antarctic sandstone rocks (Selbmann et al., 2015). The cryptoendolithic lifestyle offers, among other lithic niches, more stable conditions for microbial residents, such as protection from abiotic stresses found at the rock surface (Golubic et al., 1981; Onofri et al., 2007). The distribution of black fungi in Antarctic rocks is strictly influenced by the intensity of solar radiation, which contains high UV levels due to the local ozone depletion (Coleine et al., 2022). While *K. petricola* was mainly found in southern (shaded) cryptoendolithic communities, *C. antarcticus* indifferently inhabits both southern (shaded) and northern (sun‐exposed) cryptoendolithic communities (Selbmann et al., 2021).

Here, it was observed that *K. petricola* WT:A95—strain isolated from the Mediterranean—grows faster than *C. antarcticus* even at the low temperature of 15°C. This suggests that the fungus thrives over a wider temperature range than previously thought. Both fungi form similarly shaped colonies on the tested substrates when incubated in either constant darkness (DD) or LD, which simulated day–night changes. *C. antarcticus* WT:515, unlike *K. petricola*, secretes DHN melanin intermediates in nutrient‐rich media, which are susceptible to photooxidation. The secretion of similar metabolites in response to extra nitrogen sources has also been reported for the black fungus *Exophiala viscosa* (Carr et al., 2023). Since fungi living in extreme, oligotrophic environments have to spare energy, it is not clear why they discard such expensive metabolites. However, this apparently cost‐inefficient response may be limited to high nutrient availability in axenic conditions, as no metabolites were secreted by *C. antarcticus* in the oligotrophic mimic WA.

Microcolonial black fungi can—but unlike lichenized fungi, do not have to—interact with phototrophic algae and cyanobacteria to form functional communities. The latter require light for photosynthesis, but excessive sunlight with harmful UV can cause cell damage. In this respect, black fungi in cryptoendolithic communities and subaerial biofilms are thought to act as protective filters, by shielding the rest of the microbial community and mitigating photooxidative damage with their melanin content (Gorbushina, 2007; Nienow et al., 1988). To fulfil this role, fungal cells must be able to survive exposure to excessive sunlight while reducing the UV radiation and allowing the transmittance of wavelengths for photosynthesis. Although both *K. petricola* and *C. antarcticus* may have the ability to produce DOPA melanin, the major melanin is of the DHN type, as deletion of *pks1* abolishes melanization. The availability of non‐melanized mutants allowed us to study the role of DHN melanin for survival and protection against UV‐B irradiation in the same experimental set‐up. As anticipated, melanized *C. antarcticus* was able to survive higher UV‐B doses in single cell assays than melanized *K. petricola*. Considering that both fungi produce the same type of melanin, it was surprising that its absence had distinct consequences in the two fungi. DHN melanin does not protect *K. petricola* from UV‐B at all, whereas it contributes significantly to the UV‐B tolerance of *C. antarcticus*. This denotes that the mere presence of DHN melanin in (black) fungi does not provide equal protection. In the case of *K. petricola* and *C. antarcticus*, the amount and organization of DHN melanin in the cell wall may explain the difference in survival capabilities upon UV‐B exposure. *K. petricola* cell walls contain a thinner and therefore more flexible layer of DHN melanin compared to *C. antarcticus* cell walls (Breitenbach et al., 2022; Catanzaro et al., 2024), which is less protective but on the other hand allows for faster propagation by budding. Despite the total amount in the cell wall, the respective DHN melanin polymers may differ in how and to which cell wall components they are cross‐linked.

*K. petricola* produces high amounts of carotenoids in addition to DHN melanin, resulting in an intense pink pigmentation of the Δ*pks1* colonies. Neither the lack of carotenoids nor both pigments reduced UV‐B survival rates in *K. petricola*, suggesting that the constitutive production of carotenoids has a function other than photoprotection. As carotenoids are embedded in the membrane, they may maintain the fluidity of the lipid layer over a range of temperatures, for example, for growth at lower temperatures found in Antarctica, and for coping with desiccation stress as a consequence of higher temperatures in Mediterranean regions. Whether mycosporines contribute to UV protection in *K. petricola* is still an open question, but may be addressed in future by genetic approaches, as the synthesis pathway in fungi has recently been revealed (Sepulveda et al., 2023).

UV irradiation directly affects DNA, resulting in cytotoxic and mutagenic CPDs and 6‐4 PPs. These UV lesions can be repaired in different ways, for example, by NER, AER mediated by the UV endonuclease UVE1, or photoreactivation by photolyases. The latter light‐driven repair mechanism is highly advantageous in sun‐exposed oligotrophic environments, as it saves metabolic energy by using solar energy (UV‐A to blue fraction) for cleavage of the photodimers. Considering that solar radiation is the only natural source of UV radiation, it always provides the prerequisite for light‐driven repair. Photoreactivation, as indicated by the higher UV‐B survival rates in LD compared to DD, was observed in both species. However, the pigmented and pigment‐deficient *K. petricola* strains fully recovered from UV‐B exposure, whereas the *C. antarcticus* wild type and UV‐sensitive Δ*capks1* mutant did not. Their lower survival rates may be due to reduced photoreactivation capacities and/or a consequence of the inherent sensitivity to white light of *C. antarcticus*. Consistent with the observed ability to employ light for DNA repair, photolyase‐encoding genes were found in the genome of both fungi (Table S2). The presence of two putative CPD photolyases in *K. petricola* compared to one in *C. antarcticus* may result in a more efficient photoreactivation. Taken together, both fungi contain photolyases to repair CPDs and 6‐4 PP lesions, and also a putative UV endonuclease whose orthologs in *Neurospora crassa*, *Cryptococcus neoformans* and *B. cinerea* protect cells from UV damage by additionally removing CPDs and 6‐4 PPs (Verma & Idnurm, 2013; Yajima et al., 1995; Zhu et al., 2018). Genes encoding UV endonucleases and photolyases, as well as other photoreceptors, are usually expressed in a light‐dependent manner, ensuring that these enzymes are (only) available and functional when needed (Dasgupta et al., 2016; Schumacher, 2017; Yu & Fischer, 2019). The occurrence of photoreactivation in melanized and non‐melanized *K. petricola* and *C. antarcticus* strains is a first hint that white light enters the cells—despite the melanized cell walls—to mediate DNA repair. Accordingly, light might be sensed by the existing photoreceptors to regulate cell physiology and entrain a circadian clock.

In sum, the wild‐type strains of *K. petricola* and *C. antarcticus* exhibited different responses to white light and UV‐B. While the former maintained full cell viability when exposed to white light but had lower tolerance to UV‐B irradiation, the latter showed the inverse trend, with inhibition of cell growth under white light and higher tolerance to UV‐B. *K. petricola* shows remarkable versatility in colonizing rocks under stressful conditions, resulting in a wide distribution across temperate and desert habitats. Its defence against UV‐induced damage is photoreactivation rather than protection by melanin. When faced with elevated levels of UV‐B as in the Antarctic drylands, *K. petricola* may pursue an avoidance strategy by associating with shaded lithic communities. Conversely, *C. antarcticus* is specialized to live in the harsh Antarctic environment, and its survival strategy is primarily UV and light avoidance through the cryptoendolithic lifestyle, hyperaccumulation of DHN melanin and slow growth. As a back‐up strategy for survival, DNA photorepair may still be used by this fungus when living within sun‐exposed lithic communities.

## CONCLUSIONS

Melanins, produced by various fungi, have broadly been considered to mediate photoprotection. However, the available data are difficult to compare and interpret because of the different types of fungal melanin produced (e.g., DHN, DOPA, and pyomelanin) and the use of different cell types (e.g., spores, vegetative cells), media (e.g., availability of nutrients), and UV irradiation (e.g., UV‐A, UV‐B, or UV‐C) in these experimental studies. Black fungi, a polyphyletic group specialized in (poly)extremotolerance, exhibit reduced life cycles by producing only melanized vegetative cells. The possibility of genetically modifying their genomes now allows a comprehensive study of these fungi, starting with testing the hypothesis that (DHN) melanin protects them from solar radiation. Here, the wild type and pigment‐deficient mutants of *K. petricola* (four strains) and *C. antarcticus* (two strains) have provided the basis for a detailed mechanistic study. By finding that DHN melanin can, but does not necessarily, protect (black) fungi from UV‐B, this study highlights the importance of carrying out genetic studies across different species, rather than relying on assumptions based on limited knowledge from other fungi. Considering that UV irradiation, usually UV‐C, is used to kill unwanted microbes on surfaces, in water or in the air, knowledge of species‐specific capabilities in UV tolerance, including preferred survival strategies (e.g., mechanical by thick DHN melanin layers and/or enzymatic by light‐driven photolyases), is important to develop effective strategies to prevent the growth of black fungi that colonize sun‐exposed subaerial surfaces and may also be opportunistic pathogens colonizing surfaces in domestic and hospital environments.

## AUTHOR CONTRIBUTIONS

**Ilaria Catanzaro:** Conceptualization; methodology; writing – original draft; writing – review and editing; data curation; investigation; visualization. **Anna A. Gorbushina:** Funding acquisition; writing – review and editing; resources. **Silvano Onofri:** Funding acquisition; writing – review and editing; supervision; conceptualization. **Julia Schumacher:** Conceptualization; writing – review and editing; writing – original draft; supervision; investigation; visualization.

## CONFLICT OF INTEREST STATEMENT

The authors declare no conflicts of interest.

## Supporting information


**TABLE S1:** Oligonucleotides used in this study.
**TABLE S2:** *Knufia petricola* and *Cryomyces antarcticus* proteins putatively involved in light signalling or DNA repair.

## Data Availability

The data that supports the findings of this study are available in the supplementary material of this article.
