# Supplementary material for: 1,8‐Dihydroxynaphthalene (DHN) melanin provides unequal protection to black fungi Knufia petricola and Cryomyces antarcticus from UV‐B radiation
Source: Environ Microbiol Rep. 2024 Nov 15;16(6):e70043. doi: 10.1111/1758-2229.70043 (PMC11567843; doi:10.1111/1758-2229.70043)
Supplement: Supplementary file 1 — TABLE S1: Oligonucleotides used in this study. TABLE S2: Knufia petricola and Cryomyces antarcticus proteins putatively involved in light signalling or DNA repair. [file EMI4-16-e70043-s001.pdf]

Table S1. Oligonucleotides used in this study.

Name	Sequence (5'← 3')	Binding site
***kppks1*-SH-hi5F**	GGTTGTCGGCAGTGATACGACAAG	Upstream of *kppks1* (–1.283 to 1.259 kb)
***kppks1*-PS1sF1**	CTACGTGTTCGGTGACCAAA	In *kppks1* (+0.011 to 0.031 kb)
***kppks1*-PS1sR2**	GTTGCGCGAACGGTGCTTACC	In *kppks1* (+0.277 to 0.298 kb)
***kppks1*-sF1**	GCTCTGAGAGAAGTCATCCTCGAG	In *kppks1* (+0.928 to 0.952 kb)
***kppks1*-wtF3**	GGCCGACTGAACTACTTCTTCAAG	In *kppks1* (+1.606 to 1.630 kb)
***kppks1*-wtF2**	GCCGATCTGGCATACACCACCAC	In *kppks1* (+2.587 to 2.610 kb)
***kppks1*-wtR2**	GTCCGAGACGCCGTTGATGCATG	In *kppks1* (+3.200 to 3.223 kb)
***kppks1*-sF3**	GTCCAGCTCTACCATCAGCGAATC	In *kppks1* (+3.519 to 3.543 kb)
***kppks1*-com3F**	TGAGAACGGTGAGGCCGATAAGGTGAGCAGAAAGATGGCA	In *kppks1* (+4.830 to 4.420 kb)
***kppks1*-sF4**	GACACAGTAGAGTCCGGCATCAC	In *kppks1* (+5.341 to 5.364 kb)
***kppks1*-hi3R3**	GCGCCGTCTATAACCTTTGCAATAG	Downstream of *kppks1* (+6.858 to 6.883 kb)
***kppks1*-SH-hi3R**	GAGTTAGATTCGAGACACTCCACCAG	Downstream of *kppks1* (+7.112 to 7.138 kb)
***kpppt1*-hi5F**	GTCAAGCATTCTTGGCTCCGCG	Upstream of *kpppt1* (–0.337 to 0.315 kb)
***kpppt1*-hi3R**	GCCATCGATGTGGCCTCTAAGTG	Downstream of *kpppt1* (+1.365 to 1.388 kb)

Open reading frames: *kppks1* – 6.601 kb (one intron of 52 bp) [GenBank accession: MT859418.1], *kpppt1* – 1.101 kb (no introns) [GenBank accession: PP374627.1].

Table S2. ***Knufia petricola*** **and** ***Cryomyces antarcticus*** **proteins putatively involved in light signaling or DNA repair.**

Description	*K. petricola* proteins			*C. antarcticus* proteins		% aa identity shared by *Kp* and *Ca*
	Name	Size	GenBank acc.	JGI protein ID	Size	
**Cryptochrome photolyase family (CPF) protein |** DASH cryptochrome: UV-A/BLUE	**KpCPF1**	607 aa	**WWZ17649.1**	**Cryan3|1036121**	650 aa	**53 %**
**Cryptochrome photolyase family (CPF) protein |** CPD photolyase: UV-A/BLUE	**KpCPF2**	648 aa	**WWZ17650.1**	**Cryan3|982802**	644 aa	**50 %**
**Cryptochrome photolyase family (CPF) protein |** CPD photolyase: UV-A/BLUE	**KpCPF3**	539 aa	**WWZ17651.1**	*no hit*	n/a	n/a
**Cryptochrome photolyase family (CPF) protein |** (6-4) photolyase: UV-A/BLUE	**KpCPF4**	718 aa	**WWZ17652.1**	**Cryan3|999807**	643 aa	**50 %**
**GATA-type transcription factor, WC-1 ortholog (LOV):** BLUE	**KpWCL1**	1,069 aa	**WAK13571.1**	**Cryan3|1059543**	1,033 aa	**47 %**
**GATA-type transcription factor, WC-2 ortholog**	**KpWCL2**	517 aa	**WAK13572.1**	**Cryan3|443880**	491 aa	**51 %**
**GATA-type transcription factor, NsdD/SUB-1/LTF1 ortholog**	**KpLTF1**	466 aa	**WWA97541.1**	**Cryan3|1024422**	1,158 aa	**17 %**
**Light-oxygen-voltage (LOV) domain-containing protein:** BLUE	**KpLOV2**	1,224 aa	**WWZ17653.1**	*no hit*	n/a	n/a
**Light-oxygen-voltage (LOV) domain-containing protein:** BLUE	**KpLOV3**	749 aa	**WWZ17654.1**	**Cryan3|1085497**	595 aa	**35 %**
**Light-oxygen-voltage (LOV) domain-containing protein:** BLUE	**KpLOV4**	549 aa	**WWZ17655.1**	**Cryan3|1018934**	503 aa	**33 %**
**Light-oxygen-voltage (LOV) domain-containing protein:** BLUE	*no hit*	n/a	n/a	**Cryan3|845159**	626 aa	n/a
**Microbial opsin (OPS):** GREEN	**KpOPS1**	298 aa	**WWZ17656.1**	**Cryan3|1097610**	328 aa	**52 %**
**Microbial opsin (OPS) |** *ops2* linked with carotenogenic genes**:** GREEN	**KpOPS2**	306 aa	**QOE76782.1**	**Cryan3|563816**	322 aa	**56 %**
**Microbial opsin (OPS):** GREEN	**KpOPS3**	282 aa	**WWZ17657.1**	*no hit*	n/a	n/a
**Phytochrome (histidine kinase):** RED + FAR-RED	**KpPHY1**	1,519 aa	**WWZ17658.1**	**Cryan3|43883**	1,466 aa	**53 %**
**Frequency, circadian oscillator**	**KpFRQ1**	957 aa	**WWZ17659.1**	**Cryan3|447692**	769 aa	**23 %**
**UV endonuclease (alternative excision (dark) repair)**	**KpUVE1**	811 aa	**WZM82882.1**	**Cryan3|1064672**	785 aa	**47 %**

Putative photoreceptors of *K. petricola* were described previously (Schumacher and Gorbushina, 2020).

Those of *C. antarcticus* were identified in the database of annotated proteins of *C. antarcticus* CBS 116301 at the Joint Genome Institute (Home - *Cryomyces antarcticus* CBS 116301 v3.0 (doe.gov)) by BlastP analyses using the *K. petricola* protein sequences as queries. The proteins deduced from the primary alleles are listed.

Additional abbreviations: DASH – *Drosophila*, *Arabidopsis*, *Synechocystis*, *Homo*; CPD – cyclobutane pyrimidine dimer; (6-4) – pyrimidine-pyrimidone adduct [(6-4) photoproduct.

WC-1 and WC-2 (*white collar*) and SUB-1 (*submerged protoperithecia*) are the names of the transcription factors from *Neurospora crassa* where they were first described.
